# Metabolomics in Childhood Asthma – a Promising Tool to Meet Various Clinical Needs

**DOI:** 10.1007/s11882-025-01198-6

**Published:** 2025-05-09

**Authors:** Natalia Rzetecka, Jan Matysiak, Joanna Matysiak, Paulina Sobkowiak, Irena Wojsyk-Banaszak, Anna Bręborowicz, Kacper Packi, Agnieszka Klupczyńska-Gabryszak

**Affiliations:** 1 Department of Inorganic and Analytical Chemistry, Poznan University of Medical Sciences, Poznan, Poland; 2 Faculty of Health Sciences, Calisia University, Kalisz, Poland; 3 Department of Pulmonology, Pediatric Allergy and Clinical Immunology, Poznan University of Medical Sciences, Poznan, Poland; 4 Department of Nucleic Acid Biochemistry, Medical University of Lodz, Lodz, Poland; 5 AllerGen Center of Personalized Medicine, Piotrkow Trybunalski, Poland; 6 Wladyslaw Bieganski Collegium Medicum, Jan Dlugosz University in Czestochowa, Częstochowa, Poland

**Keywords:** Pediatric asthma, Biomarkers, Metabolites, Metabo-endotypes

## Abstract

**Purpose of Review:**

The aim of our review is to summarize the available literature where metabolomics was used in studies on childhood asthma, and to find metabolites that are diagnostic biomarker candidates in childhood asthma. Moreover, the review also describes studies related to metabo-endotypes and heterogeneity of childhood asthma, severity of the disease, and response to drug treatment.

**Recent Findings:**

Metabolomics has opened up new perspectives in childhood asthma investigation. Based on the available literature, we found nine metabolites that demonstrated the highest diagnostic potential for differentiation between children with asthma and healthy controls: adenine, adenosine, benzoic acid, hypoxanthine, p-cresol, taurocholate, threonine, tyrosine, and 1-methyl nicotinamide. Many of the identified metabolites are closely associated with inflammatory processes responsible for asthma. Metabolomic analysis also contributed to characterizing new asthma endotypes highlighting the heterogeneity of pediatric asthma.

**Summary:**

Metabolomics can bring about valuable insights, which, when integrated with other omic disciplines, can facilitate the diagnosis and management of childhood asthma and the search for new biomarkers of the disease. Improvements in the detection of asthma in preschool children, including asthma endotypes, will ease application of proper treatment and enable elimination of unnecessary test treatment of corticosteroids in young patients.

## 1. Introduction

Asthma is a heterogeneous disease, considered one of the civilization diseases. In 2022, the number of asthma patients in the world was about 300 million [1]. In 1993, the Global Initiative for Asthma (GINA) was created to disseminate the latest information and knowledge about asthma treatment and diagnosis. The organization releases an annual report to increase global awareness of asthma [2]. Asthma is an inflammatory respiratory tract disease characterized by recurring symptoms, including cough, shortness of breath, wheezing, and chest pain [3]. It is caused by a combination of genetic and environmental factors, such as pollution, stress, obesity, and smoking [4]. According to the Global Asthma Report, the incidence of asthma in children and adolescents also depends on the continent and country in which they live or migrate to, which is associated with asthma risk due to environmental changes [5].

Children with asthma represent a challenging group of patients extremely difficult to diagnose. Early diagnosis of asthma and prompt initiation of treatment allows for good disease control, prevention of its exacerbations, and progression toward the development of irreversible changes in the bronchi, i.e., remodeling. If the diagnosis is made accurately and early, the patient not only achieves symptom control faster but also has the possibility of proper psychomotor development and a higher quality of life. Other respiratory diseases presenting with the same symptoms are rare, it is common to commence asthma therapy without first excluding other possible respiratory conditions. This leads to unnecessary application of steroid treatment in all children suspected of asthma. Diagnosis of childhood asthma can be also challenging due to various types of asthma that depend on Th2 cytokines (IL-4, IL-5, IL-13), eosinophilic inflammation, allergies, exercise-induced symptoms, or aspirin-exacerbated reactions [6].

Childhood asthma diagnosis is based mainly on the patient's symptoms and clinical evaluation of symptoms or allergies, exclusion of other causes of bronchial obstruction, and responsiveness to anti-inflammatory therapy [7]. For older children, more diagnostic methods are available, such as spirometry (> 5 years), which consists of a functional test of the respiratory system, and oscillometry (> 3 years). A measurement of the fractional exhaled nitric oxide (FeNO) flow, airflow variability or bronchial provocation tests, and IgE serum tests are also performed [8]. Bronchoscopy involving collection of bronchial secretion or a sample for histopathological examination can be conducted, but it is categorized as an invasive and more expensive test [9]. Looking at phenotypic features is insufficient to provide an accurate diagnosis, and looking upstream (at metabolites and proteins) may be informative. Changes in metabolites can be crucial in diagnosing asthma, particularly when it coexists with other diseases, such as food allergies and atopic dermatitis.

Omics sciences, such as transcriptomics, epigenomics, metabolomics, and proteomics, used alone or in combination, could be a key to developing new and effective diagnostics for a variety of conditions, including childhood asthma. Metabolomics allows for the identification and quantification of low-molecular-weight compounds (metabolites) in biological samples. Metabolites serve various functions in the body, and include such groups of chemicals as amino acids, organic acids, sugars, fatty acids, lipids, steroids, small peptides, and vitamins [10, 11]. Potential metabolic biomarkers can be identified and determined using mass spectrometry – a highly sensitive and selective analytical technique. Frequently used techniques in metabolomic experiments include liquid chromatography-mass spectrometry (LC–MS), gas chromatography-mass spectrometry (GC–MS), and nuclear magnetic resonance (NMR) [12]. Using these methods, metabolomic studies of biological samples from patients with asthma symptoms can contribute to identification of metabolites associated with asthma pathogenesis, which can lead to improved understanding of underlying molecular mechanisms and to finding novel biomarkers of the disease.

The aim of this review is to summarize the available literature where metabolomics was used in studies on childhood asthma. We reviewed different types of biological samples that were the most commonly used in metabolomic studies of childhood asthma. Special attention was paid to research aimed at searching for diagnostic markers of childhood asthma, as studies focused on the prediction of pediatric asthma development have been reviewed recently [13]. We described the metabolite features with high discriminatory ability in childhood asthma detection that were identified as biomarker candidates in more than one study. Contrary to previous reviews of metabolomic studies of asthma, our review focuses solely on childhood asthma [14, 15]. Moreover, the review discusses not only research aimed at selecting metabolites with a diagnostic potential in childhood asthma but also research designed to study metabolic alterations among children with asthma to identify different asthma metabotypes/endotypes. Childhood asthma endotyping and metabotyping is increasingly discussed in the scientific literature [3, 16], and may lead to many breakthroughs in diagnostics and therapy. To complete the picture of asthma heterogeneity, we also included studies in the field of pharmacometabolomics, which is one of the latest trends in metabolomics research [17].

## 2. Literature Search

**Fig. 1:**
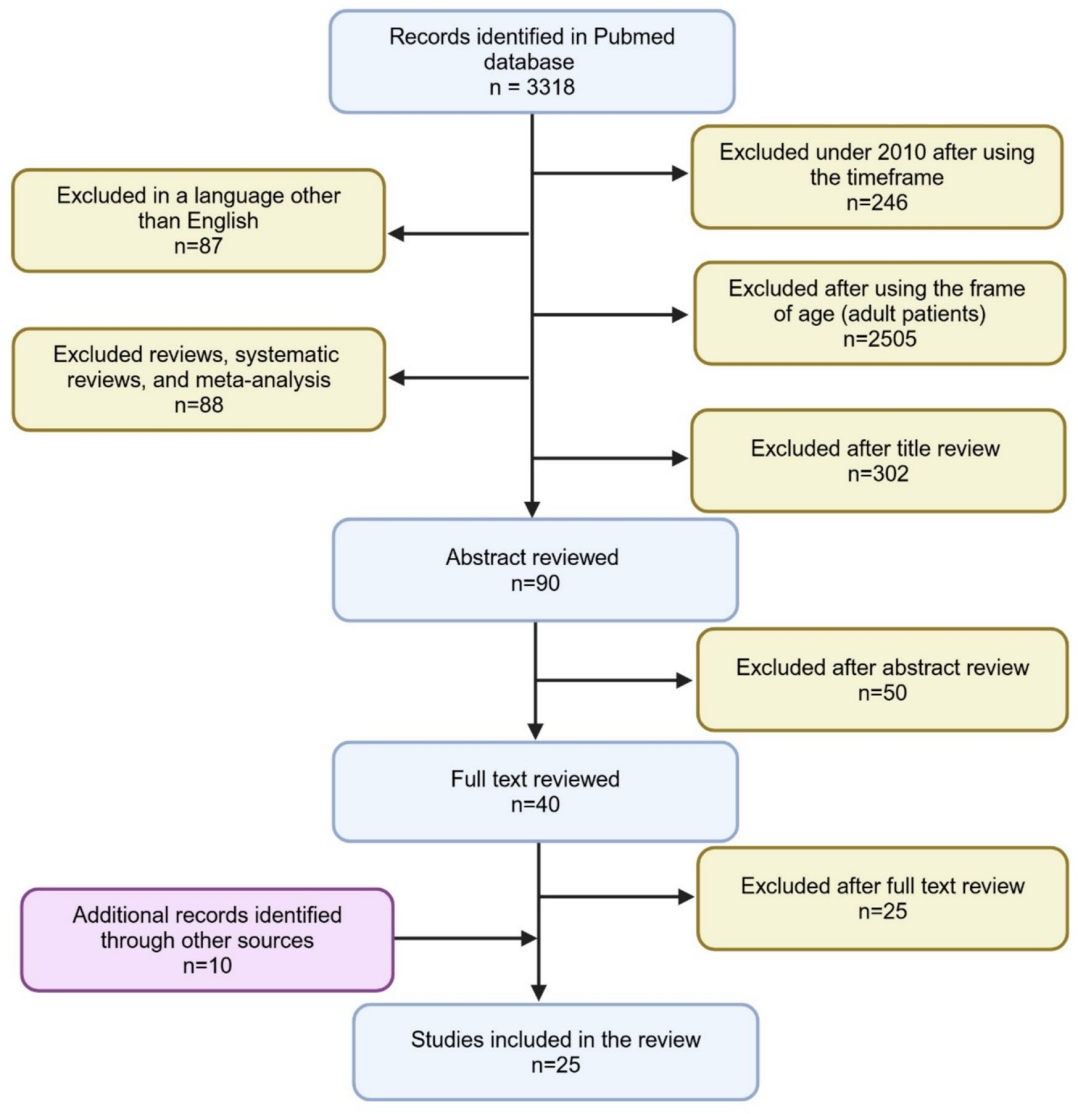
Summary of literature search for reviewed articles. Created in BioRender.com

A systematic literature search was conducted using the Pubmed (https://pubmed.ncbi.nlm.nih.gov/) database. The literature review spanned the time from January 2010 to April 2024. We decided to review articles from the last 14 years, considering significant development of metabolomics methods in this timeframe and increased interest in applying metabolomics in childhood asthma. The articles were searched using different combinations of the following keywords: “childhood asthma”, “asthma”, “children with asthma”, “asthmatic children”, “metabolomics”, “metabolomic studies”, “metabolites”, “asthma metabolites”, “asthma endotypes”, “asthma metabolic endotypes”, “endotypes”, “metabotypes”. Our literature review included studies on identification of metabolites as biomarkers from children with asthma. First, we excluded articles pertaining to the timeframe and data on adult patients as well as reviews, systematic reviews, and meta-analyses. Then, the articles were verified based on their title and abstract review. We also searched Google Scholar (https://scholar.google.com/) database and hand-searched other articles and reviews to complete the literature review. Due to the many heterogeneity in study groups, analytical methods, matrixes and study objectives in the articles found, it was not possible to perform a classic meta-analysis. The results of the literature search are shown in Fig. 1.

## 3. Selection of Biospecimens and Analytical Techniques for Metabolomic Studies of Childhood Asthma

Metabolomics allows for a large-scale qualitative and quantitative analysis of low-molecular-weight metabolites from blood (serum, plasma), saliva, urine, induced sputum, exhaled breath condensate (EBC), nasal lavage fluid, and stool samples. Sample selection is based on the goal of the study, an assumption to be proven or verified, and how easy, difficult, or invasive it is to collect [18]. During sample selection, it is important to consider the sample size required for testing and the potential for sample repeatability.

Asthma is difficult to diagnose in children. It varies in severity and frequency, and there are no standardized diagnostic methods [19]. The youngest children are challenging patients due to their lack of communication on the patient’s side. During medical examination, the best option would be to take samples in a way that is as non-invasive and quick as possible [19]. In our review, we summarized which biological samples were taken most often in childhood asthma metabolomic studies, and what their disadvantages and advantages were during an analysis.

EBC samples were used in ten out of 25 reviewed studies (Tables 1, 2). EBC as a biological sample is non-invasive to collect, but the procedure is difficult in pediatric patients, especially children under 5 years old. It is the fluid from the respiratory tract. The test involves the patient exhaling into a special device for about 10 min, allowing condensation to accumulate [20]. The condensate contains compounds that cause/reflect disorder or inflammation in the respiratory tract. Also, throughout the testing procedure, the biochemical processes taking place in the samples are not disrupted. Problematically, their intensity can be easily changed by inflammation, oxidative stress, and medication [21].

**Table 1:** Metabolomic studies of childhood asthma aimed at searching for diagnostic biomarkers

Reference	Study design	Matrix	Method	Metabolites differentiating the research and control group
**Kelly et al., 2018 **[24]	**Research group**: 46 children with asthma, **Age** (mean): 8.4 **Male/Female**: 18/28 **Control group**: 191 healthy controls **Age** (mean): 7.9 **Male/Female**: 95/96	Plasma	LC–MS/MS; GC–MS	n1-methyl-2-pyridone-5-carboxamide ↑, taurocholate ↑, biliverdin ↓, 5,6-dihydrothymine ↓, p-cresol sulfate ↑, tryptophan betaine ↓, cortisone↓, 1-docosapentaenoylglycerophosphocholine (22:5) ↓
**Checkley et al., 2016 **[25]	**Research group**: 50 children with asthma (25 normal weight, 25 overweight), **Age** (mean): 13.1 **Male/Female**: 32/ 18 **Control group**: 50 healthy controls (25 normal weight, 25 overweight) **Age** (mean): 13.9 **Male/Female**: 25/25	Serum	LC–MS	2-isopropylmalic acid ↓, ascorbic acid ↓, 6-phospho-d-gluconate ↓, shikimate-3-phosphate ↓, glutathione ↑, pyrophosphate ↓, hexose-phosphate ↑, maleic acid ↓, 2-hydroxy-2-methylbutanedioic acid ↓, hypoxanthine ↑, d-glucarate ↓, 2-oxobutanoate ↓, methylnicotinamide ↑, trehalose-6-phosphate ↑
**Matysiak et al., 2020 **[26]	**Research group**: 13 children with asthma **Age** (median^a^): 12 **Male/Female**: 7/6 **Control group**: 17 healthy controls **Age** (median^a^): 10 **Male/Female**: 11/ 6	Serum	LC–MS/MS	taurine↓, L-valine↓, DL-β-aminoisobutyric acid↓, ƴ-amino-n-butyric↑, L-arginine↑
**Crestani et al., 2020 **[27]	**Research group**: 35 children with asthma **Age** (mean): 7.5 **Male/Female**: 20/ 15 **Control group**: 20 healthy controls **Age** (mean): 6.3 **Male/Female**: 10/10	Serum	LC–MS/MS	^c^ 1-(1-enyl-palmitoyl)−2-oleoyl-GPC (P-16:0/18:1), 1-(1-enyl-oleoyl)-GPE (P-18:1), 1-(1-enyl-stearoyl)-GPE (P-18:0), 1-arachidonoyl-GPE (20:4n6), 1,2-dipalmitoyl-GPC (16:0/16:0), 1-myristoyl-2-arachidonoyl-GPC (14:0/20:4), 1-myristoyl-2-palmitoyl-GPC (14:0/16:0), 1-palmitoyl-2-stearoyl-GPC (16:0/18:0), 1-stearoyl-2-arachidonoyl-GPI (18:0/20:4), 3-hydroxyhexanoate, caproate (6:0), heptenedioate (C7:1-DC), cholesterol, oleoyl-arachidonoyl-glycerol (18:1/20:4), behenoyl dihydrosphingomyelin (d18:0/22:0), myristoyl dihydrosphingomyelin (d18:0/14:0), palmitoyl dihydrosphingomyelin (d18:0/16:0), sphingomyelin (d17:1/14:0, d16:1/15:0), sphingomyelin (d18:0/20:0, d16:0/22:0), sphingomyelin (d18:1/14:0, d16:1/16:0), ceramide (d16:1/24:1, d18:1/22:1), lactosyl-N-nervonoyl-sphingosine (d18:1/24:1), taurochenodeoxycholate, taurocholate, glycocholate, lysine, 2-aminoadipate, N2-acetyllysine, leucine, N-acetylleucine, isoleucine, valine, N-acetylvaline, 3-methylglutaconate, gamma-glutamylalanine, gamma-glutamyl-alpha-lysine, gamma-glutamylthreonine, glutamate, glutamine, N-acetylthreonine, sarcosine, threonine, methionine, N-formylmethionine, N-acetylmethionine, N-acetylhistidine, 4-hydroxyphenylpyruvate, orotate, 3-aminoisobutyrate, dimethylarginine, N-acetylneuraminate
**Chiu et al., 2020 **[28]	**Research group**: 28 children with asthma **Age** (mean): 3.6 **Male/Female**: 19/9 **Control group**: 26 healthy controls **Age** (mean): 3.6 **Male/Female**: 16/10	Plasma	NMR	histidine↑
**Saude et al., 2011 **[9]	**Research group**: 73 children with asthma (40 outpatients with asthma, 33 blind outpatients with asthma) **Age** (median^a^): 9; 10 **Male/Female**: 50/23 **Control group**: 42 healthy controls (32 healthy controls, 10 blind healthy controls) **Age** (median^a^): 8.4; 14 **Male/Female**: 26/16	Urine	NMR	1-methylhistamine↑, 1-methylnicotinamide ↓, 2-hydroxyisobutyrate ↑, 2-oxoglutarate ↑, 3-methyladipate ↑, 4-aminohippurate ↑, 4-pyridoxate↓, adenine ↑, adenosine ↑, carnitine↑, hippurate ↑, homovanillate↑, methylamine ↓, o-acetylcarnitine ↓, phenylalanine ↑, succinate ↓, threonine ↓, trigonelline ↑, trimethylamine noxide ↓, tryptophan ↑, tyrosine ↑, yellow-7.1 ↑
**Mattarucchi et al., 2011 **[29]	**Research group**: 41 children with asthma **Age** (median^a^): 11 **Male/Female**: no data **Control group**: 12 healthy controls **Age** (median^a^): age-matched to the research group **Male/Female**: no data	Urine	LC–MS	urocanic acid↓, methyl-imidazoleacetic acid↓
**Carraro et al., 2018 **[30]	**Group 1**: 16 children with early-onset asthma **Age** (mean): 4 **Male/Female**: 11/5 **Group 2**: 16 children with transient wheezing **Age** (mean): 4 **Male/Female**: 11/5	Urine	LC–MS	4-(4-deoxy-α-d-gluc-4-enuronosyl)-d-galacturonate ↑, glutaric acid ↑, 4-hydroxynonenal ↑, phosphatidyl glycerol↑, 3-methyluridine↑, steroid O-sulfate↑, 5-hydroxy-l-tryptophan↑, 3-indoleacetic acid↑, tiglylglycine↑, indole↑, cytosine↑, N-acetylputrescine↑, indole-3-acetamide↑, 6-methyladenine↑, 5-methylcytosine↑, N-acryloylglycine↑, hydroxyphenyllactic acid↑, oxoadipic acid↓, (-)-epinephrine↓, l-tyrosine↓, 3-hydroxyhippuric acid↓, benzoic acid↓, 3-hydroxy-sebacic acid↓, dihydroferulic acid 4-sulfate↓,p-cresol↓, indolelactic acid↓, N-acetyl-l-phenylalanine↓, N2-acetyl-ornithine↓
**Chiu et al., 2018 **[31]	**Research group**: 30 children with asthma **Age** (range^b^): 1–4 **Male/Female**: 9/21 **Control group**: 30 healthy controls **Age** (range^b^): 1–4 **Male/Female**: 13/17	Urine	NMR	dimethylamine↓, 1-methylnicotinamide↓, allantoin↓, guanidoacetic acid ↑
**Tao et al., 2019 **[32]	**Research group**: 80 children with asthma (37 uncontrolled, 43 controlled) **Age** (mean): 7.5 **Male/Female**: 56/24 **Control group**: 29 healthy controls **Age** (mean): 7.2 **Male/Female**: 19/10	Urine	GC–MS	uric acid↓, stearic acid↓, d-threitol↓, n-acetylgalactosamine↑, heptadecanoic acid↓, aspartic acid↑, xanthosine↑, hypoxanthine↑
**Chiu et al., 2020 **[28]	**Research group**: 28 children with asthma **Age** (mean): 3.6 **Male/Female**: 19/9 **Control group**: 26 healthy controls **Age** (mean): 3.6 **Male/Female**: 16/10	Urine	NMR	1-methylnicotinamide↓, trimethylamine N-oxide↓
**Carraro et al., 2007 **[33]	**Research group**: 25 children with asthma **Age** (range^b^): 7–15 **Male/Female**: 18/14 **Control group**: 11 healthy controls **Age** (range^b^): 7–15 **Male/Female**: 15/12	EBC	NMR	acetylated compounds, oxidized compounds
**Dallinga et al., 2010 **[34]	**Research group**: 63 children with asthma **Age** (mean): 10.8 **Male/Female**: 47/ 16 **Control group**: 57 healthy controls **Age** (mean): 9.9 **Male/Female**: 29/28	EBC	GC–MS	(branched) hydrocarbon (C13H28) ↑, carbon disulphide (CS2) ↑, 1-penten-2-on↓, butanoic acid↑, 3-(1-methylethyl)-benzene↑, (branched) hydrocarbon (C13H28) ↑, unsaturated hydrocarbon (C15H26) ↑, benzoic acid↑, p-xylene↓, (branched) hydrocarbon (C11H24) ↓
**Esther et al., 2009 **[35]	**Research group**: 14 children with asthma **Age** (mean): 10.4 **Male/Female**: 5/9 **Control group**: 35 healthy controls **Age** (mean): 11.3 **Male/Female**: 20/15	EBC	LC–MS/MS	adenosine↑, AMP↑, purine
**Carraro et al., 2013 **[36]	**Research group**: 42 children with asthma (31 with nonsevere asthma, 11 with severe asthma) **Age** (mean): 10.8 **Male/Female**: 26/ 16 **Control group**: 15 healthy controls **Age** (mean): 12.6 **Male/Female**: 7/8	EBC	LC–MS	retinoic acid↑, deoxyadenosine↑, vitamin D↑
**Caldeira et al., 2012 **[37]	**Research group**: 32 children with asthma **Age** (range^b^): 4–16 **Male/Female**: 18/14 **Control group**: 27 healthy controls **Age** (range^b^): 3–6 **Male/Female**: 15/12	EBC	GC–MS	nonane↑, 2,2,4,6,6-pentamethylheptane↑, decane, 3,6-dimethyldecane↑, dodecane↑, tetradecane↑, 6-methyl-5-hepten2-one↓, 1-dodecene↓, nonanal↓, decanal↓, dodecanal↓
**Gahleitner et al., 2013 **[38]	**Research group**: 11 children with asthma **Age** (mean): 12 **Male/Female**: 8/3 **Control group**: 12 healthy controls **Age** (mean): 12 **Male/Female**: 8/4	EBC	GC–MS	1-(methylsulfanyl)propane↑, ethyl benzene↑, 1,4-dichlorobenzene↑, 1,7-dimethylnapthalene↑, 2-octenal↑, octadecyne↑, 1-isopropyl-3-methylbenzene↑, 4-isopropenyl-1-methylcyclohexene↑
**Smolińska et al., 2014 **[39]	**Research group**: 76 children with asthma **Age** (mean): 3.1 **Male/Female**: 45/31 **Control group**: 49 healthy controls **Age** (mean): 3.3 **Male/Female** 24/25	EBC	GC–MS	acetone↓, 2,4-dimethylheptane↑, 2,4-dimethylpentane↑, 2,2,4-trimethylheptane↓, 1-methyl-4-(1-methylethenyl) cyclohexen ↓, 2,3,4-trimethyloctane↓, 2-undecenal↑, biphenyl↓, 2-ethenylnaphtalene↓, 2,6,10-trimethyldodecane↓, octane↑, 2-methylhexane↑
**Chang-Chien et al., 2020 **[40]	**Research group**: 92 children with asthma **Age** (mean): 6.3 **Male/Female**: 49/43 **Control group**: 73 healthy controls **Age** (mean): 6.4 **Male/Female**: 37/36	EBC	NMR	lactate↑, formate↑, butyrate↑, isobutyrate↑
**Ferraro et al., 2020 **[41]	**Research group**: 26 children with asthma **Age** (mean): 9.1 **Male/Female**: 20/6 **Control group**: 16 healthy controls **Age** (mean): 10.2 **Male/Female**: 11/5	EBC	LC–MS	9-amino-nonanoic acid↑, 12-amino-dodecanoic acid↑, lactone of PGF-MUM↑, N-linoleoyl taurine↑, 17-phenoxy trinor PGF2α ethyl amide↑, lysoPC (18:2(9Z,12Z)) ↑
**Tian et al., 2017 **[42]	**Research group**: 20 children with asthma **Age** (mean): childhood **Male/Female**: no data **Control group**: 15 healthy controls **Age** (mean): childhood **Male/Female**: no data	Sputum	LC–MS	1-hexadecanoyl-sn-glycerol↓, glycerol 1-stearate↓, sphingosine↓, phe-ser↑, tyr-ala↑, phe-gln↑, cytidine 2’,3’-cyclic phosphate↓, 1-hexadecanoyl-2-(9Z-octadecenoyl)-sn-glycero-3-phospho-(1’-rac-glycerol) ↓, 1-octadecanoyl-2-(9Z-octadecenoyl)-sn-glycero-3-phosphoserine↓, thymidine↑, gamma-L-glutamyl-L-valine↑, adenine↑

Abbreviations *EBC* exhaled breath condensate, *GC–MS* gas chromatography-mass spectrometry, *LC–MS* liquid chromatography-mass spectrometry, *LC–MS/MS* liquid chromatography-tandem mass spectrometry, *NMR* nuclear magnetic resonance

^a^ median value (mean values were not provided in all publications)

^b^ range (mean values were not provided in all publications)

^c^ metabolites significantly different (p < 0.005) in pairwise comparisons of children with asthma and healthy controls

↑ higher levels in the research group than in the control group

↓ lower levels in the research group than in the control group

The underlined sentence means: correlated significantly (p < 0.05) with the ICS dose that the asthmatic patients were on as regular treatment

**Table 2:** Metabolomic studies on heterogeneity of childhood asthma – studies related to metabo-endotypes of childhood asthma, severity of the disease, and response to drug treatment

Reference	Study design	Matrix	Method	Main goal	Main findings
**Quan-Jun et al., 2017 **[43]	**Group 1**: 69 children with asthma during acute exacerbation who had not been treated with a corticosteroid or a beta2-agonist in the previous three months., receiving inhaled budesonide and salbutamol for at least five treatments depending on the disease severity and patient age, **Age** (mean): 4.7 **Male/Female**: 35/ 34 **Group 2**: 48 children with asthma without the previous usage of any corticosteroids or beta2 agonists, **Age** (mean): 5.2 **Male/Female**: 22/26	Serum	NMR	The aim of the study was to determine the effect of budesonide and salbutamol (glucocorticoid and beta2-agonist) on the metabolic profile of children with asthma	Higher level in group 1: 4-hydroxybutyrate, lactate, cis-aconitate, 5-HIAA, taurine, trans-4-hydroxy-l-proline, tiglylglycine, 3-hydroxybutyrate, 3-methylhistidine, glucose Higher level in group 2: alanine, glycerol, arginine, glycylproline, 2-hydroxy-3-methylvalerate, creatine, citrulline, glutamate, asparagine, 2-hydroxyvalerate, citrate
**Rago et al., 2021 **[44]	**Discovery group**: children with and without asthma (577 aged 6 months and 513 aged 6 years) **Age** (mean): 6 months and 6 years **Male/Female**: 294/283 **Validation group**: 469 children followed for asthma/recurrent wheezing until the age of 3 years **Age** (mean): 1 year **Male/Female**: 273/240	Plasma	LC–MS/MS	The aim of this study was to study the endotype of sphingolipid-associated childhood asthma using multiomics data	The findings indicate the existence of a sphingolipid-associated childhood asthma endotype, characterized by an early onset of symptoms and increased airway resistance by the age of six years
**Kelly et al., 2022 **[16]	**Discovery group**: 1151 children with asthma **Age** (mean): 9.2 **Male/Female**: 682/ 469 **Validation group**: 911 children with mild to moderate asthma **Age** (mean): 12.9 **Male/Female**: 549/ 362	Plasma	LC–MS	The aim of the study was to validate metabolomics-based asthma endotypes	Five metabo-endotypes of asthma with different characteristics, such as cholesterol esters, triglycerides, and fatty acids are proposed based on the results
**Quan-Jun et al., 2017 **[43]	**Group 1**: 69 children with asthma during acute exacerbation who had not been treated with a corticosteroid or a beta2-agonist in the previous three months., receiving inhaled budesonide and salbutamol for at least five treatments depending on the disease severity and patient age, **Age** (mean): 4.7 **Male/Female**: 34/ 35 **Group 2**: 48 children with asthma without the previous usage of any corticosteroids or beta2 agonists, **Age** (mean): 5.2 **Male/Female**: 22/26	Urine	NMR	The aim of the study was to determine the effect of budesonide and salbutamol (glucocorticoids and beta2-agonists) on the metabolic profile of children with asthma	Higher level in group 1: cis-aconitate, lactate, 2-deoxyinosine, 3-methylhistidine, 5-HIAA 2-aminoadipate, glucose Higher level in group 2: citrulline, homoserine, histamine, alanine, asparagine, glycylproline, sn-glycero-3-phosphocholine, sarcosine, ornithine, creatine, creatinine, glycine, isoleucine, trimethylamine N-oxide
**Park et al., 2016 **[45]	**Group 1**: 15 corticosteroid responders (CS-R) children with asthma **Age** (mean): 12.8 **Male/Female**: 11/ 4 **Group 2**: 15 corticosteroid nonresponders (CS-NR) children with asthma **Age** (mean): 13.9 **Male/Female**: 10/5	Urine	LC–MS	The goal of this study was to further understand corticosteroid resistance among children with severe asthma	Higher level in group 1: γ-glutamylcysteine Higher level in group 2: cysteine-glycine
**Fitzpatrick et al., 2014 **[46]	**Asthma group 1**: 22 children with mild-to-moderate asthma **Age** (mean): 12 **Male/Female**: 12/10 **Asthma group 2**: 35 children with severe refractory asthma **Age** (mean): 13 **Male/Female**: 23/12	EBC	LC–MS	The aim of this study was to research the metabolomic profile of mild-moderate than severe asthma	The results indicate that glycine, serine, and threonine can be associated with corticosteroid refractory asthma in children

Abbreviations: *EBC* exhaled breath condensate, *LC–MS* liquid chromatography-mass spectrometry, *LC–MS/MS* liquid chromatography-tandem mass spectrometry, *NMR* nuclear magnetic resonance

Urine is another biological material that was used in metabolomic studies of children with asthma. As indicated in Tables 1 and 2, urine was utilized in eight out of the 25 studies reviewed. Urine is a biological fluid that can be collected and re-collected in an easy and non-invasive way. It has a less complex composition and contains less protein than blood. The composition of urine can vary depending on the gender and age of the patient [10]. Due to a simple collection procedure from young children, urine remains a commonly utilized specimen in metabolomic studies. However, in contrast to EBC or saliva, urine is not directly related to the respiratory tract.

Eight of the studies listed in Tables 1 and 2 included blood (plasma or serum) samples. Blood has a well-described composition and contains molecules secreted from all over the body. Blood samples are attractive research matrices due to a potentially large number of metabolites that can be determined. Such samples allow for determination of metabolites responsible for various physiological conditions in the body. These compounds include hormones, albumin, globulins, enzymes, lipids, and amino acids [22]. Importantly, blood can be analyzed using various techniques and methods, and their collection is minimally invasive [18].

Sputum used to be frequently utilized as a biological sample for diagnostic purposes. This biological material is rarely utilized in asthma metabolomics, and it was used in one of 25 studies reviewed in this article. The material is related to the respiratory tract. As with other biological samples, it has a well-known methodology for collection and analysis. However, sputum samples can be easily contaminated with saliva and red blood cells [23], and are very difficult to obtain in young children.

In summary, our review of 25 studies examining metabolites in childhood asthma showed that the most commonly utilized biological samples were EBCs (10 articles). There were fewer samples of urine (8 articles), blood (8 articles), and sputum (1 article). LC–MS was the most common analytical technique used (14 articles), while NMR (8 articles), and GC–MS (6 articles) were used less frequently (Table 1, Table 2).

The type of biological sample collected in the metabolomic studies affects the choice of the analytical method used for metabolic profiling. This review of the literature (Tables 1 and 2) revealed that GC–MS and LC–MS were the most frequently utilized methods for EBC testing, LC–MS for blood samples analysis, whereas NMR and LC–MS for urine samples analysis. Other factors affecting the choice of analytical method include a research question, compounds of interest, and equipment availability. It should be borne in mind that the selection of methods for metabolomic studies affects the obtained results, and no analytical technique provides coverage of all metabolites contained in a sample. Therefore, the most complete metabolite profile is yielded by a combination of results from different analytical platforms [24].

## 4. Metabolomic Searching for Diagnostic and Predictive Biomarkers of Childhood Asthma

### 4.1. Study Design

In the research summarized in Table 1, the study group consists of children with asthma, and the control group consists of healthy children without asthma or its symptoms. Metabolomic studies of childhood asthma differed in terms of the patient’s age and sample size. In the study group, the age of the children with asthma ranged from 0 to 18 years (Table 1). The lowest mean age in the study group was 3.1 years [39], and the highest 13.1 years [25]. The lowest and highest mean age in the control group was 3.3 and 13.9 years, respectively [25, 39]. In five out of the 20 articles (Table 1), studies were conducted on samples collected from children up to 5 years of age [28, 30, 31, 37, 39]. Most patients in the study and control groups were male. Male patients with asthma accounted for 62%, and female patients with asthma accounted for 38% of the total population from Table 1. The proportion of healthy male patients was 54%, while that of healthy female patients was 46%. Worth highlighting is the fact that the study groups outnumbered the control groups (Table 1). The size of the research group ranged from 11 to 92 children [38, 40]. The size of the control group ranged from 11 to 191 individuals [24, 33]. The study performed by Kelly et al. [24] utilized the largest patient population with a sample size of 237 patients (Table 1). The smallest study population of 23 patients was investigated by Gahleitner et al. [38]. Saude et al. [9] employed external validation using 33 blinded samples from outpatients with asthma and ten blinded healthy controls. In our review, we looked at metabolic differences between healthy and pediatric asthma patients based on 20 articles on this topic. Our goal was to find metabolites with promising diagnostic potential and define their possible functions in the body related to children asthma.

### 4.2. Metabolites with the Highest Diagnostic Potential in Childhood Asthma

**Fig. 2:**
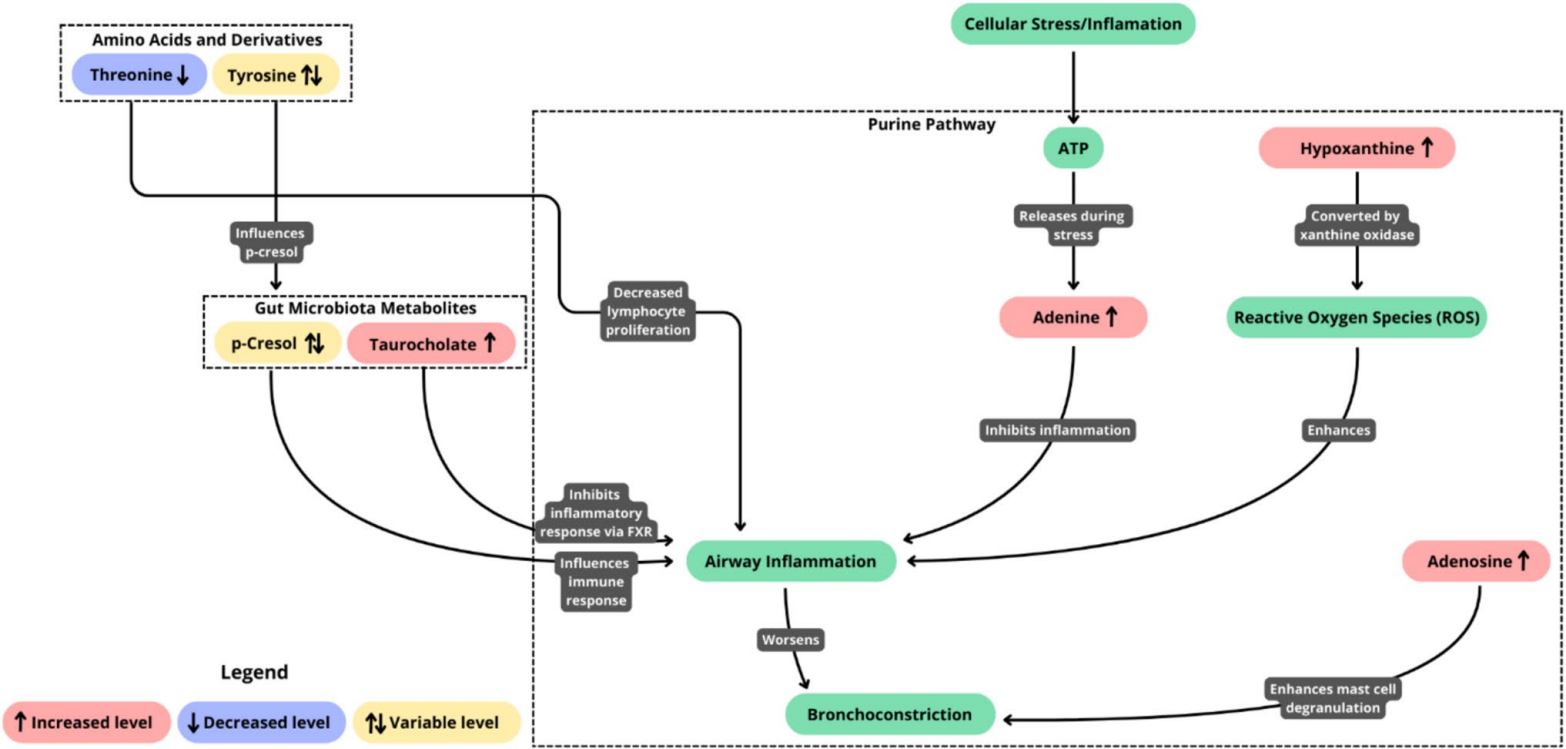
Metabolic pathways and their involvement in childhood asthma pathogenesis based on performed metabolomic studies. The diagram illustrates the interactions between key metabolites identified as discriminative features in childhood asthma metabolomic studies. Three main metabolic groups are highlighted: the purine pathway (including adenine, adenosine, and hypoxanthine), gut microbiota metabolites (p-cresol and taurocholate), and amino acids with their derivatives (threonine and tyrosine). Arrows indicate metabolic relationships and physiological effects. Cellular stress initiates ATP release, leading to increased adenine levels, which shows anti-inflammatory properties. Elevated adenosine levels enhance mast cell degranulation, contributing to bronchoconstriction. Hypoxanthine, through xanthine oxidase activity, generates reactive oxygen species (ROS), exacerbating airway inflammation. Gut microbiota-derived metabolites demonstrate variable effects: p-cresol shows inconsistent levels across studies, while increased taurocholate exhibits anti-inflammatory properties through FXR activation. Among amino acids, decreased threonine levels may impair lymphocyte proliferation and tyrosine shows variable levels affecting p-cresol production. Color coding indicates metabolite level changes in asthmatic children: pink (↑) represents increased levels, blue (↓) indicates decreased levels, and yellow (↑↓) shows variable levels reported across different studies. Green boxes represent physiological processes

Metabolomic studies of pediatric asthma identified numerous metabolites that differentiate between children with asthma and those without asthma (Table 1). In the articles included in the review, nine metabolites were identified as significant discriminative features in two or more studies: adenine, adenosine, benzoic acid, hypoxanthine, p-cresol, taurocholate, threonine, tyrosine, and 1-methyl nicotinamide (Table 3). Although the clinical utility of the above-mentioned compounds remains unclear and has to be proved in further studies, they can increase our understanding of the molecular mechanisms of childhood asthma. Metabolites and their interactions in the pathogenesis of childhood asthma and their possible association with airway inflammation were included in the pathway analysis (Fig. 2).

**Table 3:** Metabolites identified as relevant in two or more childhood asthma metabolomic studies aimed at searching for diagnostic biomarkers

Metabolite	Class of metabolite	Subclass of metabolite	Type of matrix	Level of metabolites in asthma patients	Article references
Adenine	Imidazopyrimidines	Purines and purine derivatives	Urine	↑^a^	Saude et al., 2011 [9]
			Sputum	↑	Tian et al., 2017 [42]
Adenosine	Purine nucleosides	Not available	Urine	↑	Saude et al., 2011 [9]
			EBC ^c^	↑	Esther et al., 2009 [35]
			EBC	↑	Carraro et al., 2013 [36]
Benzoic acid	Benzene and substituted derivatives	Benzoic acids and derivatives	EBC	↑	Dallinga et al., 2010 [34]
			Urine	↓^b^	Carraro et al., 2018 [30]
Hypoxanthine	Imidazopyrimidines	Purines and purine derivatives	Serum	↑	Checkley et al., 2016 [25]
			Urine	↑	Tao et al., 2019 [32]
p-Cresol	Phenols	Cresols	Plasma	↑	Kelly et al., 2018 [24]
			Urine	↓	Carraro et al., 2018 [30]
Taurocholate	Steroids and steroid derivatives	Bile acids, alcohols and derivatives	Plasma	↑	Kelly et al., 2018 [20]
			Serum	No data	Crestani et al., 2020 [27]
Threonine	Carboxylic acids and derivatives	Amino acids, peptides, and analogues	Serum	No data	Crestani et al., 2020 [27]
			Urine	↓	Saude et al., 2011 [9]
Tyrosine	Carboxylic acids and derivatives	Amino acids, peptides, and analogues	Urine	↑	Saude et al., 2011 [9]
			Urine	↓	Carraro et al., 2018 [30]
1-Methyl nicotinamide	Pyridines and derivatives	Pyridinecarboxylic acids and derivatives	Urine	↓	Saude et al., 2011 [9]
			Urine	↓	Chiu et al., 2018 [31]
			Urine	↓	Chiu et al., 2020 [28]

^a^ higher levels in the research group than in the control group, ^b^ lower levels in the research group than in the control group, ^c^ EBC exhaled breath condensate

Adenine is one of five nitrogenous bases of DNA and RNA. It is involved in the metabolism of purines. It is a component of adenosine triphosphate (ATP) and adenosine diphosphate (ADP), and is, therefore, essential for energy storage and transfer in various cellular processes [47]. Extracellular ATP is released during cellular stress and injury, leading to the activation of purinergic receptors, which subsequently promotes the release of pro-inflammatory cytokines [48]. Metabolomic research showed increased adenine levels in the group of children with asthma in comparison with the control group. Elevated levels of adenine may be indicative of disorders of the purine metabolism. Adenine was shown to inhibit LPS-induced inflammation in mouse macrophage cells, primary mast cells, and peritoneal cells and attenuate inflammatory cytokine release [49].

Adenosine is accumulated in cells in response to metabolic stress or damage. In patients with asthma, adenosine is often associated with pro-inflammatory effects [50]. This is due to its ability to induce bronchoconstriction and enhance mast cell degranulation [51]. Higher adenosine levels were found in children with asthma than those without asthma in three metabolomic studies (Table 3). The application of different analytical techniques in those studies suggests a high diagnostic potential of adenosine and the possibility of its robust determination using different methods. Increased adenosine levels in asthmatic children suggest its involvement in ongoing inflammation and hypoxia-related responses in asthma [52].

Benzoic acid is widely used as a preservative in food, feed, and pharmaceutical industries due to its strong antimicrobial properties [53, 54]. Two metabolomic studies found Benzoic acid as a childhood asthma-discriminating metabolite (Table 3). Interestingly, in EBC samples [34], it occurred at higher concentrations in asthma patients than in controls, while in urine samples [30], a reverse relationship was shown. The identification of xenobiotics as potential markers raises questions about their origin in the biofluids and their clinical utility. Therefore, the altered levels of benzoic acid in childhood asthma should be interpreted with caution.

Hypoxanthine belongs to the purine metabolite group. It is converted to uric acid by xanthine oxidase, a process that generates reactive oxygen species (ROS) [55]. These ROS contribute to oxidative stress, which is a key feature of chronic inflammatory diseases such as asthma. Increased levels of hypoxanthine were reported in serum and urine collected from children with asthma (Table 3). Markedly higher level of hypoxanthine was also found in adult patients with asthma [55]. Elevated hypoxanthine levels are indicative of increased oxidative stress and hypoxia, which are commonly observed in asthmatic patients during exacerbations [56]. This means that hypoxanthine levels may serve as a marker of asthma severity in children.

p-Cresol is an aromatic amino acid metabolism metabolite produced by human and animal gut microflora. It binds to proteins and, as a partially lipophilic molecule, can be strongly associated with plasma proteins [57]. p-Cresol is excreted with urine, and its concentration in the plasma may be indicative of kidney health or dysfunction. p-Cresol levels were found higher in plasma samples of children with asthma and in lower levels in urine samples of children with asthma (Table 3). Different directions of changes in this metabolite level can result from different biofluids analyzed and various characteristics of the enrolled patients. Elevated levels of p-cresol in patients with asthma could indicate changes in the gut microbiota, which are known to influence systemic inflammation and immune responses. This highlights the potential role of gut microbiota and its metabolic products in the pathogenesis of asthma and suggests that targeting these pathways might offer new therapeutic approaches [58].

Taurocholate is found in the small intestine, supporting digestion and absorption of fats and vitamins, and is synthesized in the liver from cholesterol [59]. Along with other bile acids, can interact with immune cells and influence the production of cytokines, which are crucial in managing inflammation and airway reactivity in asthmatic patients. Studies have demonstrated that taurocholate can inhibit inflammatory responses by activating specific receptors, such as the farnesoid X receptor (FXR) [60]. Taurocholate was found in higher levels in children with asthma in a study by Kelly et al. [29]. Crestani et al. [27] also found a difference between the research and control groups, however, it did not provide information on the direction of change. They also pointed to differences in taurocholate levels in patients with asthma and those with various allergic and atopic diseases. The elevated levels of taurocholate may suggest its involvement in reducing inflammation and preventing excessive immune responses, which are hallmarks of asthma pathophysiology [60, 61].

Threonine is an essential amino acid, and it needs to be supplemented with food participating in lipid metabolism and protein synthesis. Threonine is needed for the production of collagen, and elastin plays a role in maintaining a healthy appearance of the skin [62]. More importantly, threonine plays relevant functions in the immune system, where lymphocytes use it to support their proliferation and secretion of antibodies [62]. It is a part of the gastrointestinal mucin and is also responsible for intestinal immune function. Saude et al. [10] found lower threonine concentrations in the children with asthma than in the control group. It can be supposed that lower threonine levels in young patients with asthma may be due to the development of asthma-induced inflammation.

Tyrosine is an endogenous amino acid. Its presence affects catecholamine neurotransmitters, such as dopamine and norepinephrine. It influences energy levels, improves memory, and shows significant cognitive benefits. Tyrosine is metabolized by gut bacteria into p-cresol sulfate [63]. Studies in mice demonstrated that p-cresol or tyrosine can alleviate allergic diseases of the airways. Metabolomic studies showed discrepancy in urine tyrosine levels in children with asthma (Table 3). We suspect that this may be due to different age of the children patients, older than 5 years [9] and younger than 5 years [30] (Table 1). Therefore, due to the research outcome inconsistency, we cannot conclude whether lower or higher levels of tyrosine can be helpful in distinguishing patients with asthma.

1-Methyl nicotinamide is a metabolite of nicotinamide produced in the liver. This metabolite has anti-inflammatory and anti-bacterial properties [64]. 1-Methyl nicotinamide was identified as a differentiating metabolite in three urine-based metabolomic studies of childhood asthma [9, 28, 31] (Table 3). All works demonstrated lower levels of 1-methyl nicotinamide in children with asthma than in the control group. A low concentration of 1-methyl nicotinamide in urine may indicate vitamin B3 deficiency, as 1-methyl nicotinamide is a metabolite of nicotinamide. Studies in murine animal models indicate that 1-methyl nicotinamide can prevent asthma exacerbations [64]. A decrease in 1-methyl nicotinamide may be due to higher energy requirements due to asthma-caused inflammation or could indicate its deficient anti-inflammatory activity, contributing to heightened inflammatory state observed in asthma [65, 66].

Based on the information above, the metabolites identified as molecules with the highest diagnostic potential in childhood asthma demonstrate notable differences in their functions within the human body. Many of the metabolites identified as potential diagnostic markers for childhood asthma are closely linked to inflammatory processes, which underscores their potential role as biomarkers in this disease and contributes to understanding its pathophysiology. To prove the potential clinical use and robustness of these metabolites, cohort studies would have to be conducted. It is also necessary to conduct research focused on the population of preschool children, as they are the group that is the most difficult to diagnose. We suggest that utilization of targeted metabolomics techniques in the studies would assess the relevance of the proposed metabolites.

## 5. Metabolomic investigations of heterogeneity of childhood asthma

### 5.1. Phenotypes, Endotypes, and Metabotypes of Childhood Asthma

Asthma exhibits significant heterogeneity in its clinical manifestations, pathophysiological mechanisms, and treatment responses [3, 67]. This variability is captured by the concepts of phenotypes, endotypes, and metabotypes, providing a comprehensive framework for understanding asthma's complexity [6]. Phenotypes refer to observable characteristics of asthma that arise from the interaction of genetic, environmental, and lifestyle factors [68]. Endotypes delve deeper, defining subtypes based on specific pathophysiological mechanisms and molecular profiles [3]. Metabotypes, identified through metabolomic profiling, represent the metabolic foundations of asthma phenotypes and endotypes. Recognizing these distinctions is crucial for personalizing asthma management and improving patient outcomes [6].

Phenotypes of childhood asthma encompass various clinical, physiological, morphological, and biochemical symptoms unique to each patient. Influenced strongly by inherited traits, environmental factors, and demographics, phenotypes can affect asthma exacerbations depending on where a child lives. Key phenotypes include allergic and non-allergic asthma [68], eosinophilic and non-eosinophilic asthma [69], aspirin-sensitive asthma [70], exercise-induced asthma [71], and obesity-related asthma [72]. These categories underscore the importance of accurate diagnosis and tailored treatment strategies based on symptom severity [73–76].

Moving to endotypes, we encounter subtypes signifying specific disease etiologies and pathophysiological mechanisms. Endotypes at the genome, transcriptome, proteome, or metabolome levels can be identified using 'omics' techniques [6]. The well-known inflammatory endotypes in asthma include T2-high (eosinophilic), neutrophilic, pauci-granulocytic, and mixed granulocytic asthma, with childhood asthma commonly involving Th2 low and Th2 high endotypes [77].

Metabolomics identifies specific endotypes of childhood asthma, known as metabotypes, offering insights into metabolic disturbances associated with asthma and helping to tailor personalized treatment plans. Children with mild asthma exhibit less severe metabolic disturbances, stable metabolic profiles, and minimal inflammatory markers, correlating with well-controlled asthma managed with low-dose inhaled corticosteroids. Moderate asthma shows intermediate metabolic changes and inflammation, with frequent symptoms requiring regular controller medications. In severe asthma, significant metabolic disturbances and high levels of inflammatory markers are observed, with children often experiencing poorly controlled symptoms and frequent exacerbations, necessitating advanced therapies including biologics targeting specific cytokines [8, 13, 78, 79].

Recognizing and understanding phenotypes, endotypes, and metabotypes is essential for personalizing childhood asthma diagnosis and treatment. By integrating metabolomic data with clinical and physiological information, healthcare providers can develop more effective, individualized treatment plans that address the specific metabolic disturbances in each patient, ultimately improving outcomes for children with asthma.

### 5.2. Metabolomic Investigations Related to Metabo-endotypes of Childhood Asthma

In light of the pediatric asthma heterogeneity linked to clinical symptoms, disease severity, treatment response, and prognosis, various metabolomic studies were designed and performed in order to look for hints and answers to unmet clinical needs. Table 2 shows a comparison of research employing different groups of young asthma patients.

The metabolomic study of childhood asthma endotypes was a substudy performed in a cohort study by Rago et al. [44]. One of the cohorts was incorporated in the discovery group, and the second cohort was incorporated in the validation group. The study ultimately included 577 and 513 patients aged 6 months and 6 years in the discovery group, respectively, and 469 patients in the validation group. The study found that one metabolite, ceramideglycosyl-n-stearoyl-sphingosine, increased with age in children with asthma. They showed that SNP 17q21 is associated with the occurrence of asthma in children and is related to increased transcription of *ORMDL3*, which is involved in sphingolipid synthesis. However, the study did not identify a specific metabolite that could potentially become a biomarker for childhood asthma, but suggests the existence of an early-onset endotype related to sphingolipid levels.

The objective of the study by Kelly et al. [16] was to identify and validate metabo-endotypes of asthma and differences in asthma-relevant phenotypes. The study of children between 5 and 14 years of age recruited in two cohort studies. The authors identified five asthma metabo-endotypes in the first cohort, which were subsequently validated in the second cohort. Metabo-endotype group classification depended on the levels of cholesterol esters, triglycerides, and fatty acids. This was one of the largest cohort studies based on biological samples from children with asthma, so the data have great informational and diagnostic potential that can be tested in future studies based on selected metabolites.

### 5.3. Metabolomic investigations related to response to drug treatment among children with asthma

Investigations of metabolic markers related to response to treatment belong to an emerging subdiscipline of metabolomics called pharmacometabolomics [17]. Pharmacometabolomics can provide molecular information on the underlying biology of different responses to therapeutics in asthma patients [80]. Childhood asthma heterogeneity requires therapies tailored to the patients, which is in line with the precision medicine strategy.

Quan-Jun et al. [43] performed metabolomic studies on two biological fluids from children with asthma—serum and urine. The study groups were divided into two subgroups (Table 2). In total, they identified 33 differential metabolites in serum and urine analyses, and nine metabolites were recurring—lactate, cis-aconitate, 5-HIAA, 3-methylhistidine, glucose, alanine, creatine, citrulline, and asparagine. The direction of changes in recurring metabolites was the same in both biospecimens in children from both research groups. The authors also indicated that combined treatment with budesonide and salbutamol causes changes in the metabolome in children. In contrast, using them individually had no effect on the metabolomic profile in children with asthma during an acute exacerbation of the disease. This study may contribute to the understanding of the effects of different corticosteroids or beta2-agonists on the metabolomic profile in children with asthma.

Park et al. [45] conducted a urine metabolomic investigation involving two groups of children with asthma: corticosteroid responders (CS-R) and corticosteroid nonresponders (CS-NR). The study aimed to test the characteristics that differed between the groups, and 30 differences were found. The significantly altered metabolites in the CS-R and CS-NR groups included two metabolites of glutathione (GSH), γ-glutamylcysteine increased in the CS-R group and cysteine-glycine increased in the CS-NR group. The authors proposed five candidate markers to identify differences between the groups that are needed for future metabolomics studies, such as 3,6-dihydro nicotinic acid, 3-methoxy-4-hydroxyphenyl(ethylene)glycol, 3,4-dihydroxy-phenylalanine, γ-glutamylcysteine, and cysteine-glycine.

Fitzpatrick et al. [46] compared metabolites in a group of children with mild-to-moderate asthma on corticosteroid treatment and children with severe refractory asthma treated with higher doses of a corticosteroid. The study revealed that severe asthma in children is associated with a multitude of metabolic disorders, which are related to oxidative stress. The authors showed that severe, corticosteroid refractory asthma in children can be related to metabolites such as glycine, serine, and threonine. They suggest that severe asthma may be associated with other diseases, such as obesity or obstructive sleep apnea, which can be important in required further studies.

This overview presents studies in which the drugs taken and the response to them are of significant interest. They conclude that the use of different corticosteroids or beta2-agonists leads to changes in the metabolomic profile in children with asthma. These studies form the basis for future research looking for changes in the metabolomic profile caused by corticosteroid treatment.

## 6. Future perspectives and clinical needs

**Fig. 3:**
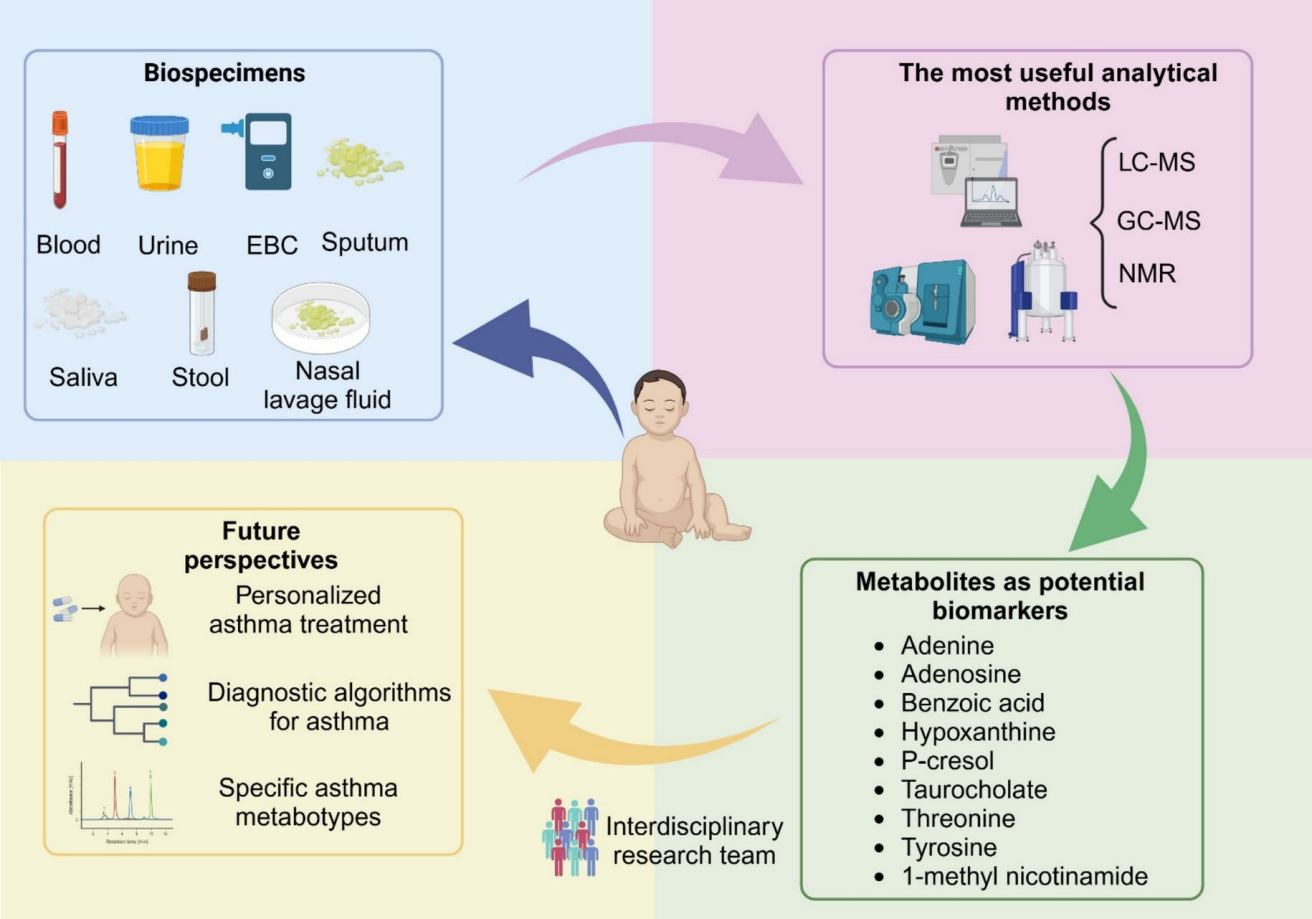
Overview illustrating possible clinical utility of metabolomic studies in childhood asthma. Created with BioRender.com

Metabolomics opened up new perspectives in childhood asthma investigation in various scientific disciplines, such as analytical chemistry, pharmacy, or molecular biology. Metabolomic investigations may lead to development of new diagnostic tools for asthma, contribute to characterizing new asthma endotypes, and accelerate implementation of personalized asthma treatment (Fig. 3). Introduction of new pediatric asthma metabo-endotypes will allow clinicians to select appropriate treatment and monitor response to the applied therapy. This is especially important in the youngest patients, when the disease begins at the stage of intensive child development.

The metabolomic research conducted so far shows promising results and indicates several metabolites that should be further studied as childhood asthma markers (Table 3). Future investigations are needed to properly assess the robustness of these marker candidates and their clinical utility. Studies employing different biofluids should be undertaken to select the most appropriate biological specimens for determination of a given compound. Among 25 studies included in the review, only two employed two various biological specimens [28, 43]. Chiu et al. [28] analyzed plasma and urine samples collected from children with asthma and a healthy control group, whereas Quan-Jun et al. [43] analyzed serum and urine collected from children with asthma during acute exacerbation and control asthma participants. The metabolomic investigation in pediatric asthma is characterized by a vast spectrum of biological samples subjected to analysis (Fig. 3). Table 3 shows good agreement or lack of agreement in the fluctuations of metabolite levels determined in different body fluids of children with asthma. Therefore, the analysis of different biofluids collected from the same group of patients, using the same analytical methodology, will provide a broader context for the observed metabolic shifts in childhood asthma, and will allow for the selection of the most informative biospecimens relevant to the intended application.

Another future direction in childhood asthma metabolomics is the inclusion of various control groups. A vast majority of the performed studies, aimed to identify diagnostic markers of pediatric asthma, employed a control group of healthy children (Table 1). However, asthma in children often coexists with allergies and other atopic diseases. Therefore, studies involving different comparative groups (e.g. children with allergy but without asthma) are needed to better interpret the obtained metabolomic data and link them to the molecular mechanisms of the disease Moreover, they allow for determination of specificity of the proposed asthma markers.

We believe that metabolomics can contribute valuable insights that, when integrated with other disciplines, will prove indispensable for the diagnosis of asthma in children. In addition, it would help to quickly differentiate asthma, respiratory diseases, or other comorbidities, such as allergy or atopic dermatitis. This is necessary to make a precise diagnosis and implement personalized treatment for young patients with asthma. It would be necessary to combine all data on childhood asthma in the specialized fields of genomics, transcriptomics, and metabolomics. So, to gather all the information together, it is essential to integrate the interconnected information points: cellular interactions, molecular relations, and biochemical mechanisms in one place as a specific disease map [81].

In the future, metabolomics can play a major role in the selection of an appropriate therapy tailored to individual patients and identification of molecular targets for new treatment strategies. It is anticipated that the number of pharmacometabolomic investigations in pediatric asthma will increase providing candidates for predictive biomarkers useful in making treatment decisions.

Asthma endotyping, particularly metabo-endotyping, is an emerging field in asthma research that leverages detailed analysis of metabolic profiles to classify asthma patients into distinct subgroups. This approach offers new avenues for understanding the disease's complexity and improving patient care. The future of asthma treatment lies in personalized medicine, where therapies are tailored to the individual's metabolic profile. By continuously refining metabo-endotyping techniques and integrating them with clinical data, researchers can develop more effective treatment plans that address unique metabolic disturbances in each patient. This personalized approach is expected to enhance treatment efficacy, reduce adverse effects, and improve overall patient outcomes and quality of life [82].

Implementation of metabolomic methods into clinical practice will require establishing of standardized protocols for analytical performance and data analysis. It should be remembered that both pre-analytical and analytical factors can influence the acquired data [83]. Therefore, standardization of sample collection, handling, and storage conditions constitute essential issues helping to preserve sample stability and obtain reproducible results [84].

## 7. Conclusions

The metabolomic studies included in the review provided fresh insight into the pathogenesis of childhood asthma, the effects of applied drugs, and the existence of asthma subtypes. We believe that the current review will benefit the design of future metabolomic studies. As experience is gained, more clinically relevant findings will likely be obtained in investigations of metabolic shifts in pediatric asthma patients. Undoubtedly, metabolomics and its subdisciplines have great translational potential for the diagnosis, treatment, and management of children with asthma. In the face of high prevalence of asthma in the pediatric population and the need for rapid implementation of treatment, it is crucial to have reliable asthma indicators. Improvements in the detection of asthma in preschool children, including asthma endotypes, will ease application of proper treatment and enable elimination of unnecessary test treatment of corticosteroids in suspected patients. They will also help decide whether to start treatment after the first episode of asthma.

## Key References

- Ferraro VA, Zanconato S, Carraro S. Metabolomics Applied to Pediatric Asthma: What Have We Learnt in the Past 10 Years? Children (Basel). 2023;10:1452. 10.3390/children10091452.
  - In this article, the authors summarized analyzes of metabolomics studies over the last 10 years in pediatric asthma, which focus on predicting asthma development, endotype characterization, and pharmacometabolomics. The article shows how the importance of metabolomics is increasing in the study of asthma in children.
- Kelly RS, Mendez KM, Huang M, Hobbs BD, Clish CB, Gerszten R, et al. Metabo-Endotypes of Asthma Reveal Differences in Lung Function: Discovery and Validation in Two TOPMed Cohorts. Am J Respir Crit Care Med. 2022;205:288–99. 10.1164/rccm.202105-1268OC.
  - The article includes a cohort study on a large population of children, and is one of the largest studies using metabolomics for asthma endotyping. In the article, the authors proposed 5 potential metabo-endotypes of asthma for more precise asthma management strategies.
- Chiu C-Y, Cheng M-L, Chiang M-H, Wang C-J, Tsai M-H, Lin G. Metabolomic Analysis Reveals Distinct Profiles in the Plasma and Urine Associated with IgE Reactions in Childhood Asthma. J Clin Med. 2020;9:887. 10.3390/jcm9030887.
  - In the article, the authors analyzed plasma and urine samples collected from children with asthma and a healthy control group aged between 3 and 5 years old. The analysis of different biofluids collected from the same group of patients provides a broader context for the observed metabolic shifts in childhood asthma.

## Data Availability

No datasets were generated or analysed during the current study.
